# The E3 ubiquitin ligase MARCH1 mediates downregulation of plasma membrane GABA_B_ receptors under ischemic conditions by inhibiting fast receptor recycling

**DOI:** 10.1038/s41598-025-85842-1

**Published:** 2025-01-08

**Authors:** Musadiq A. Bhat, Mohammad Hleihil, Irene Mondéjar, Thomas Grampp, Dietmar Benke

**Affiliations:** 1 Institute of Pharmacology and Toxicology, University of Zurich, Winterthurerstrasse 190, CH-8057 Zurich, Switzerland; 2 Neuroscience Center Zurich, University and ETH Zurich, Winterthurerstrasse 190, 8057 Zurich, Switzerland

**Keywords:** MARCH1, GABA_B_ receptor, Ubiquitination, Interfering peptide, Cerebral ischemia, Trafficking, Diseases of the nervous system, Stroke

## Abstract

GABA_B_ receptors mediate prolonged inhibition in the brain and are important for keeping neuronal excitation and inhibition in a healthy balance. However, under excitotoxic/ischemic conditions, GABA_B_ receptors are downregulated by dysregulated endocytic trafficking and can no longer counteract the severely enhanced excitation, eventually triggering neuronal death. Recently, we developed interfering peptides targeting protein-protein interactions involved in downregulating the receptors. Treatment with these peptides restored GABA_B_ receptor expression after an ischemic insult and thereby inhibited neuronal overexcitation and progressive neuronal death. In this study, we searched for GABA_B_ receptor interactions that specifically occur under ischemic conditions. We found that the E3 ubiquitin ligase MARCH1 is specifically upregulated under ischemic/excitotoxic conditions. Upregulated MARCH1 interacts with GABA_B_ receptors and triggered downregulation of plasma membrane GABA_B_ receptors by inhibiting fast recycling of the receptors. We developed an interfering peptide that inhibits the MARCH1/GABA_B_ receptor interaction. Treatment of cultured neurons subjected to ischemic stress with this peptide restored receptor expression and as a consequence stopped progressive neuronal death. Thus, inhibiting the interaction of GABA_B_ receptors with MARCH1 to restore cell surface receptor expression might be a promising strategy to prevent progressive neuronal death induced by ischemic conditions.

**Supplementary Information:**

The online version contains supplementary material available at 10.1038/s41598-025-85842-1.

## Introduction

A precise balance between neuronal excitation and inhibition is fundamental to proper brain function. In various neurological disorders, the excitation/inhibition balance is disturbed resulting most commonly in over-excitation of neurons. Prolonged neuronal over-excitation triggers detrimental pathways leading to excitotoxic neuronal death. One important component controlling neuronal excitation is the GABA_B_ receptor^1^. GABA_B_ receptors are heterodimeric G-protein coupled receptors (GPCR) consisting of the two subunits GABA_B1_ (exists as GABA_B1a_ and GABA_B1b_ variants) and GABA_B2_^2^. GABA_B_ receptors are expressed at pre- and postsynaptic locations in most neurons^3^. The binding of the neurotransmitter GABA to GABA_B_ receptors activates G_i/o_-proteins and induces prolonged neuronal inhibition by regulating the activity of several effector systems. In this regard, the most prominent ones are the activation of G protein-coupled inwardly rectifying potassium (GIRK or Kir3) channels at postsynaptic sites causing the hyperpolarization of the neuronal membrane and the inhibition of voltage-gated Ca^2+^ channels at presynaptic sites which reduces neurotransmitter release^4^.

In many neurological diseases with a disturbed excitation/inhibition balance - like addiction, anxiety, depression, neurodegenerative diseases, and cerebral ischemia - GABA_B_ receptors are downregulated from the plasma membrane^5–8^. This withdraws an important contributor to neuronal inhibition, who normally ensures that neurons do not shift into a state of over-excitation. The mechanisms responsible for the pathological downregulation of plasma membrane GABA_B_ receptors identified so far are caused by deviations from normal trafficking pathways of the receptors. Under physiological conditions, GABA_B_ receptors are constitutively internalized and most receptors are recycled to the plasma membrane while a fraction is degraded in lysosomes^9^. The degraded receptors are then replaced by newly synthetized receptors exported from the endoplasmic reticulum (ER). Pathological conditions leading to neuronal over-excitation, such as cerebral ischemia, are associated with ER stress which induces the expression of the pro-apoptotic transcription factor CHOP. Upregulated CHOP bind to the GABA_B2_ subunit in the ER, prevents their heterodimerization with the GABA_B1_ subunit and thereby the exit of the heterodimeric receptor complex from the ER^10^. This mechanism inhibits the supply of new receptors and plasma membrane downregulates GABA_B_ receptor expression because constitutively degraded receptors cannot be replaced. In addition, constitutively internalized GABA_B_ receptors are dephosphorylated at GABA_B2_(S783) by protein phosphatase 2 A (PP2A), which inhibits fast recycling of internalized receptors to the plasma membrane^11,12^. Subsequent phosphorylation of GABA_B1_(S867/T872) mediated by calcium calmodulin-dependent kinase II β (CaMKIIβ) and extracellular-signal regulated kinases 1 and 2 (ERK1/2) triggers the sorting of the receptors to the lysosomal degradation pathway^13,14^. Preventing the interaction of GABA_B_ receptors with either CHOP, CaMKIIβ or PP2A using interfering peptides restored plasma membrane receptor expression and normal GABA_B_ receptor mediated inhibition after ischemic/excitotoxic stress, resulting in the inhibition of progressive neuronal death^12,15,16^. Hence, interfering with the interaction of disease relevant GABA_B_ receptor interactions might be promising strategy for the development of specific therapeutic interventions. In case the interactions are specifically associated with the disease state, it might even be possible to selectively target the receptor population in diseased neurons. This should minimize unwanted side effects.

In the search for new protein-protein interactions that might be selectively occurring under pathological conditions and are involved in the downregulation of plasma membrane GABA_B_ receptors, we screened for ubiquitin E3 ligases that affect the cell surface expression of the receptors. We focused on E3 ligases because the expression, trafficking and degradation of many GPCRs had been shown to be regulated by ubiquitination, which often acts as master signal to decide the fate of proteins to specific cellular destinations^17^. Ubiquitination of a target protein involves an enzymatic cascade consisting of E1 (ubiquitin-activating enzyme), E2 (ubiquitin-conjugating enzyme) and E3 ligase enzymes, where the E3 ligase largely determines the substrate specificity. Hence, we screened various transmembrane E3 ligases for their ability to affect GABA_B_ receptor cell surface expression. We concentrated on transmembrane E3 ligases because they most likely reside in the same cellular compartment(s) as the receptors. We found that the E3 ligase MARCH1 (membrane-associated RING CH-type finger 1) downregulated cell surface expression of the receptors when co-expressed with GABA_B_ receptors in HEK293 cells. We characterized the effect of MARCH1 on GABA_B_ receptors and developed an interfering peptide (M1-Pep) that inhibits the interaction of GABA_B_ receptors with MARCH1. M1-Pep inhibits the interaction of GABA_B_ receptors with MARCH1 after ischemic stress, restored cell surface expression of the receptors and inhibited progressive neuronal death in cultured cortical neurons.

## Results

### Screening for transmembrane RING domain E3 ligases regulating GABA_B_ receptor expression

**Fig. 1:**
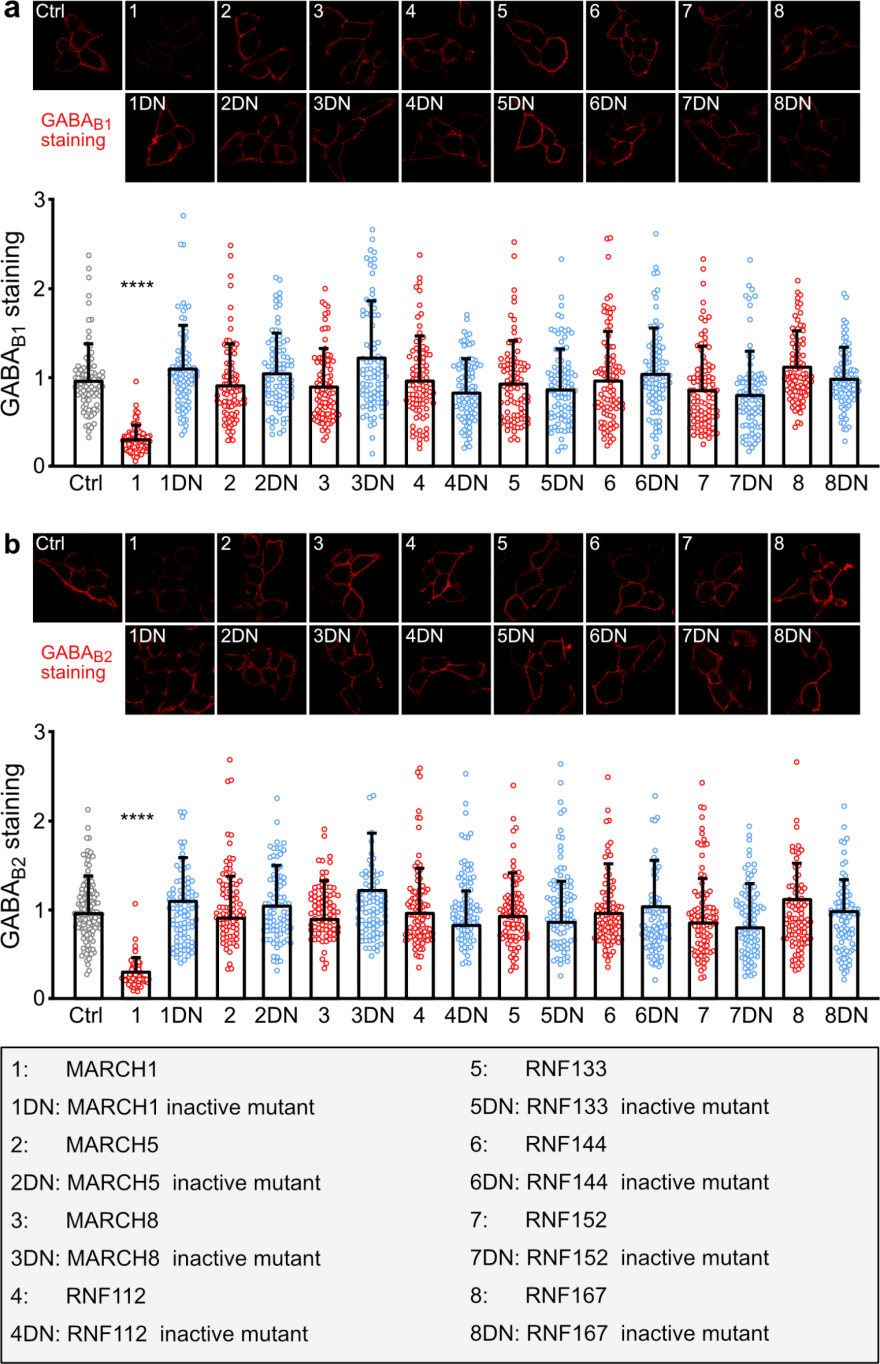
Screening for transmembrane RING domain E3 ligases regulating GABA_B_ receptor cell surface expression. HEK-293 cells were transfected with plasmids for GABA_B1_, GABA_B2_ and EGFP as a control (Ctrl) or with GABA_B1_, GABA_B2_ and the indicated E3 ligases. After 2 days, the cells were tested for cell surface expression of GABA_B1_ (**a**) and GABA_B2_ (**b**) by immunofluorescence staining using antibodies directed against GABA_B1_ and GABA_B2_. (**a**) Co-expression of MARCH1 downregulated the cell surface expression of GABA_B1_.Top: representative images (scale bar: 10 μm). Bottom: quantification of fluorescence intensities (mean ± SD of 85–90 cells per condition, 4 independent experiments). (**b**) Co-expression of MARCH1 downregulated the cell surface expression of GABA_B2_. Top: representative images (scale bar: 10 μm). Bottom: quantification of fluorescence intensities (mean ± SD of 80–132 cells per condition, 4 independent experiments). Brown-Forsythe and Welch’s one-way ANOVA followed by Games-Howell’s multiple comparisons test (****, *p* < 0.0001).

Transmembrane RING domain E3 ubiquitin ligases have been shown to regulate the cell surface expression of membrane proteins such as AMPA receptors^18^, insulin receptors^19^, CD86^20^ and major histocompatibility complex class II (MHC II)^21^. Therefore, we screened various transmembrane RING domain E3 ligases (MARCH1, MARCH5, RNF112, RNF144, RNF152, RNF167^18^, MARCH8^22^ and RNF133^23^) for their ability to affect the cell surface expression of GABA_B_ receptors. For this, we transfected HEK-293 cells with GABA_B1_ and GABA_B2_ subunits along with wildtype E3 ligases or with their corresponding functionally inactive mutants. The cell surface expression of GABA_B1_ and GABA_B2_ was determined by immunofluorescence staining using antibodies directed against the extracellular located N-terminal domains of the receptor subunits. Among the tested transmembrane RING domain E3 ligases, only the co-expression of MARCH1 affected the cell surface expression of GABA_B_ receptors. Co-expression of wildtype MARCH1 severely downregulated GABA_B1_ (Fig. 1a) and GABA_B2_ (Fig. 1b), whereas the inactive MARCH1 mutant (MARCH1(DN)) had no effect on GABA_B_ receptor cell surface expression. These results indicated that MARCH1 is involved in regulating cell surface expression of GABA_B_ receptors presumably by ubiquitination.

### MARCH1 downregulated cell surface expression of GABA_B_ receptor by mono- or multi-monoubiquitination

**Fig. 2:**
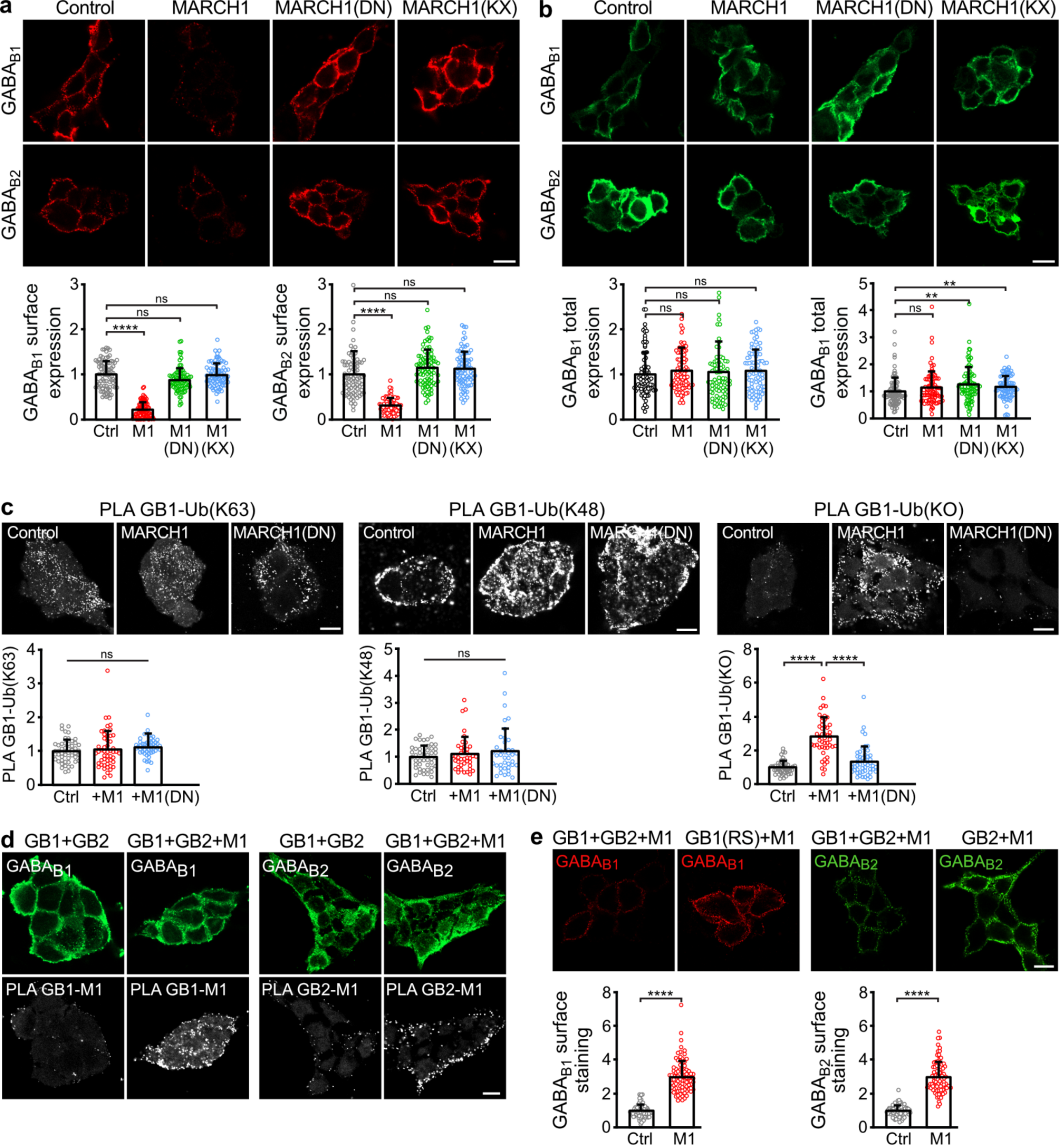
MARCH1 induced downregulation of cell surface GABA_B_ receptors requires functional MARCH1 located outside the ER and mono/multi-monoubiquitination. (**a**, **b**) HEK-293 cells transfected with GABA_B1_, GABA_B2_ and EGFP as a control (Ctrl) or with GABA_B1_, GABA_B2_ and wildtype MARCH1 (M1) or with the non-functional mutant MARCH1(DN) or the mutant MARCH1(KKXX) that cannot leave the ER were tested for cell surface (**a**) or total receptor expression (**b**). Top: representative images (scale bar: 10 μm). Bottom: quantification of fluorescence intensities (mean ± SD of 84–85 cells per condition, 3 independent experiments). Kruskal-Wallis test followed by Dunn’s multiple comparisons test (ns, *p* > 0.05; **, < 0.01; ****, *p* < 0.0001). (**c**) MARCH1 mono/multiubiquitinates GABA_B_ receptors. HEK-293 cells expressing GABA_B1_/GABA_B2_/EGFP as a control (Ctrl) or GABA_B1_/GABA_B2_/MARCH1 or GABA_B1_/GABA_B2_/MARCH1(DN) with or without a mutant of HA-tagged ubiquitin that cannot form polyubiquitin chains (Ub(KO)) were tested for ubiquitination of GABA_B_ receptors by in situ PLA using antibodies directed against GABA_B1_ and Lys63 polyubiquitin or Lys48 polyubiquitin or HA. Top: representative images (in situ PLA signals: white dots, scale bar: 10 μm). Bottom: quantification of in situ PLA signals (mean ± SD of 40–50 cells per condition, 2 independent experiments). Lys48: one way ANOVA followed by Tukey’s multiple comparison test; Lys63 and Ub(KO): Brown-Forsythe and Welch’s one-way ANOVA followed by Games-Howell’s multiple comparisons test (ns, *p* > 0.05; ****, *p* < 0.0001). (**d**) MARCH1 interacts with GABA_B_ receptors. HEK-293 cells expressing GABA_B1_/GABA_B2_ (control for non-specific PLA signal (Ctrl)) or GABA_B1_/GABA_B2_/MARCH1 were tested for interaction by in situ PLA using antibodies directed against MARCH1 and GABA_B1_ or GABA_B2_ (top images: total GABA_B_ staining, bottom images: PLA signals; scale bar: 5 μm). The images shown are representative for 90 cells analyzed per condition from 3 independent experiments. **(e)** MARCH1 does not affect the cell surface expression of single GABA_B_ receptor subunits. HEK-293 cells were transfected with MARCH1/GABA_B1_/GABA_B2_ as a control (Ctrl) or with MARCH1/GABA_B2_ or with MARCH1 and a GABA_B1_ mutant that can leave the ER (GABA_B1_(RSAR)) and tested for cell surface expression of GABA_B1_ and GABA_B2_. Top: representative images (scale bar: 10 μm). Bottom: quantification of fluorescence intensities (mean ± SD of 75 cells per condition, 3 independent experiments). Unpaired t-test with Welch’s correction (****, *p* < 0.0001).

To gain more insights into the mechanism of MARCH1 mediated downregulation of cell surface GABA_B_ receptors, we transfected HEK-293 cells with GABA_B1_ and GABA_B2_ subunits along with wildtype MARCH1 or with its corresponding mutants MARCH1(DN) and MARCH1(KKXX). MARCH1(DN) is a dominant negative mutant of MARCH1, in which conserved histidine residues within the RING domain were replaced by tryptophan, preventing zinc coordination and thereby ubiquitin ligase activity^18^. MARCH1(KKXX) lacks 40 amino acids at the C-terminus, creating a classical C-terminal di-lysine ER-retention motive, which prevents MARCH1 to leave the ER^24^. Co-expression of GABA_B_ receptors with MARCH1(DN) did not affect cell surface expression of the receptors, suggesting that E3-ligase activity – i.e. ubiquitination – is required for downregulating the receptors (Fig. 2a). Similarly, co-expression of GABA_B_ receptors with MARCH1(KKXX) did not affect cell surface expression of GABA_B1_ and GABA_B2_ (Fig. 2a), indicating that MARCH1 induces downregulation of cell surface GABA_B_ receptors in compartments outside the ER, e.g. the plasma membrane, early endosomes, recycling endosomes or Golgi apparatus. Interestingly, MARCH1 downregulated the surface expression of GABA_B_ receptors (Fig. 2a) without affecting the total expression of GABA_B1_ and GABA_B2_ (Fig. 2b). Thus, MARCH1 is not primarily be involved in the degradation pathway of GABA_B_ receptors.

Next, we tested whether MARCH1 ubiquitinates GABA_B_ receptors. For this HEK-293 cells were transfected with GABA_B1_ and GABA_B2_ along with wild type MARCH1 or the dominant negative mutant MARCH1(DN) and tested for Lys63 -and Lys48-polyubiquitination - which are the most common types of polyubiquitination - by in situ PLA using selective antibodies. However, MARCH1 did not enhance Lys63- and Lys48-polyubiquitination of GABA_B_ receptors (Fig. 2c). Then, we tested whether MARCH1 may mediate mono/multi-monoubiquitination by which a single ubiquitin moiety is attached to one (monoubiquitination) or multiple (multi-monoubiquitination) lysine residues in the target protein. For this, we used the same experimental setup described above but in addition co-transfected a mutant of ubiquitin (ubiquitin(KO)) in which all lysine residues were mutated so that it is unable to build polyubiquitin chains. Under control conditions (only GABA_B_ receptors transfected) and upon transfection with the inactive MARCH1 mutant (MARCH1(DN)) we detected only very few dots, which most likely represented nonspecific PLA signals. However, in the presence of functional MARCH1 numerous PLA signals were observed, indicating that MARCH1 most likely mediates mono- or multi-monoubiquitination of the receptors (Fig. 2c).

It is reasonable to assume that MARCH1 need to associate with GABA_B_ receptors for their ubiquitination. Using in situ PLA, we therefore tested whether MARCH1 interacts with GABA_B_ receptors when co-expressed in HEK-293 cells. As expected, co-expression of MARCH1 led to numerous PLA signals, suggesting the direct or indirect interaction of MARCH1 with the receptor (Fig. 2d).

We then tested whether MARCH1 affects the expression of GABA_B1_ or GABA_B2_ subunits when expressed alone without its counterpart. For this, HEK-293 cells were transfected with MARCH1 and either GABA_B1_(RSAR), in which the ER retention signal was mutated to allow its exit from ER and cell surface expression in the absence of GABA_B2_, or with GABA_B2_. Interestingly, MARCH1 does not appear to substantially affect cell surface expression of GABA_B1_ in the absence of GABA_B2_ and vice versa (Fig. 2e). This finding suggests that MARCH1 only affects the expression of the assembled GABA_B_ receptor heterodimer.

### MARCH1 was upregulated under ischemic conditions in neurons

**Fig. 3:**
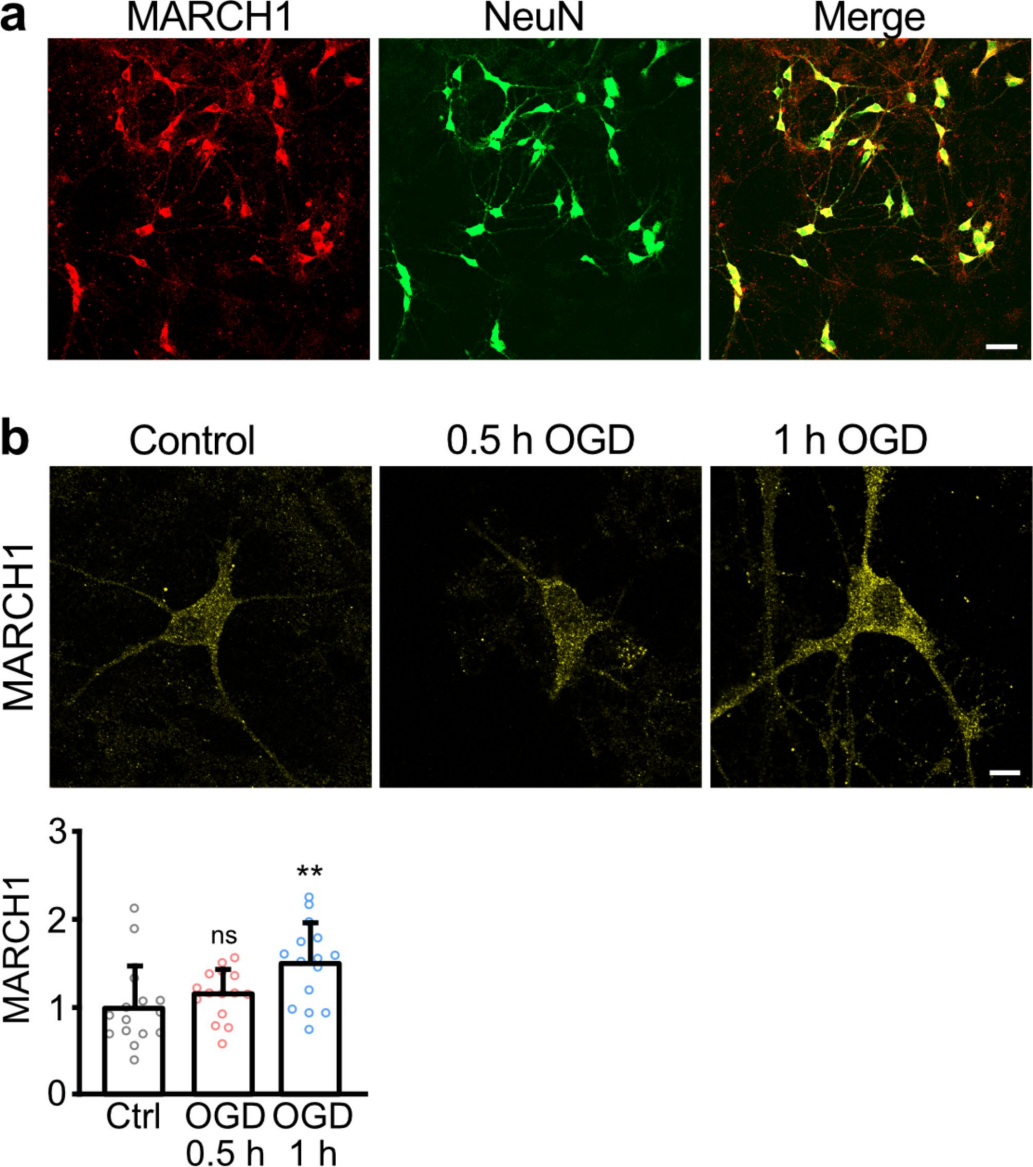
MARCH1 is expressed in neurons and is upregulated under ischemic conditions. (**a**) All neurons tested expressed MARCH1. The expression of MARCH1 in neuron/glia co-cultures was tested by immunofluorescence staining using antibodies directed against NeuN (marker protein for neurons) and MARCH1 (scale bar: 50 μm). (**b**) MARCH1 is significantly upregulated after 1 h of OGD. Neuron/glia co-cultures were subjected to OGD for 30 min–1 h and tested for MARCH1 expression by immunofluorescence staining using antibodies directed against MARCH1. Cultures not treated with OGD served as a control. Top: representative images (scale bar: 10 μm). Bottom: quantification of fluorescence intensities (mean ± SD of 15 cells per condition, 2 independent experiments). One-way ANOVA followed by Dunnett’s multiple comparison test (ns, *p* > 0.05; **, *p* < 0.01).

MARCH1 mRNA has been found to be highly expressed in spleen and lung tissue and comparatively less in muscle, liver, and brain^25,26^. To verity the expression of MARCH1 protein in neurons, we co-stained neuron/glia cultures with antibodies directed against MARCH1 and the neuron-specific marker protein NeuN (Fig. 3a). We found that all neurons tested also expressed MARCH1 signals (1074 neurons analyzed). Thus, MARCH1 appears to be ubiquitously expressed in neurons.

GABA_B_ receptors are significantly downregulated under ischemic/excitotoxic conditions^11–16,27,28^. Hence, it is plausible that upregulation of MARCH1 under ischemic condition might contribute to this pathological pathway. To test for this, we subjected cultured neurons to ischemia-like oxygen and glucose deprivation (OGD) for different time intervals and evaluated the expression of MARCH1 by immunofluorescence staining. The expression of MARCH1 was significantly upregulated after 1 h of OGD (Fig. 3b). Hence, the upregulation of MARCH1 could be involved in the pathway downregulating the GABA_B_ receptors under ischemic conditions.

### Identification of interfering peptides for restoring MARCH1 induced downregulation of cell surface GABA_B_ receptors

**Fig. 4:**
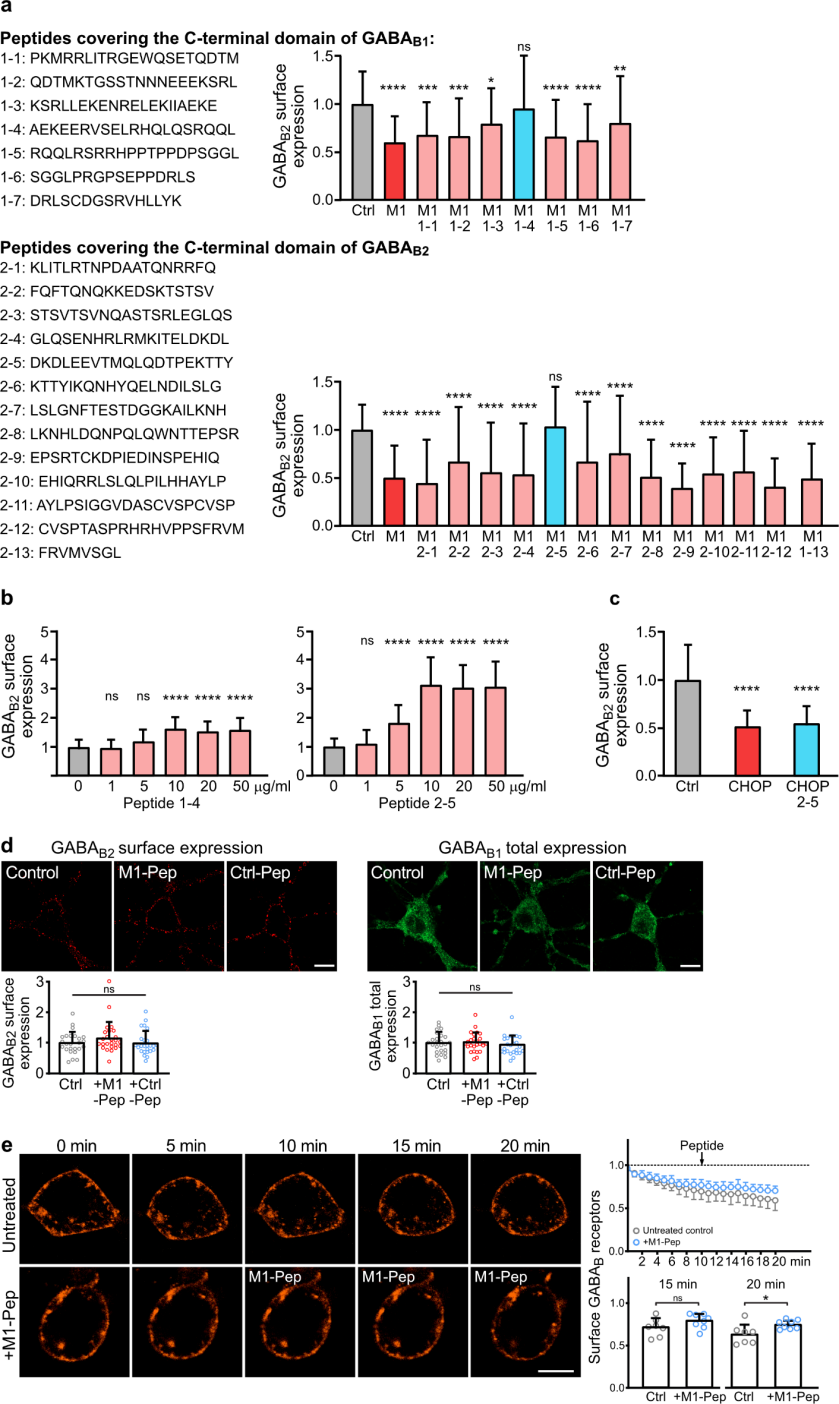
Identification of an interfering peptide for restoring MARCH1 induced downregulation of plasma membrane GABA_B_ receptors. (**a**) Screening for interfering peptides. HEK 293-cells expressing GABA_B1_/GABA_B2_ as a control (Ctrl) or GABA_B1_/GABA_B2_/MARCH1 were treated with the indicated peptides (10 µg/ml) and tested for cell surface expression of receptors using antibodies directed against and GABA_B1_ and GABA_B2_. Peptides 1–4 and 2–5 restored the cell surface expression of the receptors. Left: sequences of peptides used for screening derived from the C-terminal domains of GABA_B1_ and GABA_B2_. Right: quantification of fluorescence intensities (mean ± SD of 40–112 cells per condition, 2 independent experiments). Kruskal-Wallis test followed by Dunn’s multiple comparison test (ns, *p* > 0.05; *, *p* < 0.05; **, *p* < 0.01 ***, *p* < 0.001; ****, *p* < 0.0001). (**b**) Dose-response of peptides 1–4 and 2–5. HEK-293 cells expressing GABA_B1_/GABA_B2_/MARCH1 were treated with increasing concentrations of the respective peptides and were tested the following day for cell surface expression of GABA_B2_. The data represents the mean ± SD of 30 cells per condition from 2 independent experiments. One-way ANOVA followed by Dunnett’s multiple comparison test (ns, *p* > 0.05; ****, *p* < 0.0001) **(c)** Peptide 2–5 did not restore the CHOP mediated downregulation of GABA_B_ receptors. HEK-293 cells expressing GABA_B1_/GABA_B2_ as a control (Ctrl) or GABA_B1_/GABA_B2_/CHOP were treated with peptide 2–5 (10 µg) and tested for cell surface expression of GABA_B2_ the next day. The data represents the mean ± SD of 93–103 cells per condition from 3 independent experiments. Brown-Forsythe and Welch’s one-way ANOVA followed by Games-Howell’s multiple comparisons test (****, *p* < 0.0001) (**d**) Treatment of neurons with M1-Pep does not affect cell surface expression of native GABA_B_ receptors under physiological conditions. Neuron/glia co-cultures were treated for 16 h with or without M1-Pep or Ctrl-Pep and tested for cell surface (using GABA_B2_ antibodies) and total GABA_B_ receptor (using GABA_B1_ antibodies) expression. Top: representative images (scale bar: 10 μm). Bottom: quantification of fluorescence intensities (mean ± SD of 25 cells per condition, 2 independent experiments). One-way ANOVA followed by Tukey’s multiple comparison test (ns, *p* > 0.05). (**e**) M1-Pep marginally affects constitutive internalization of GABA_B_ receptors. Neurons transfected with GABA_B2_ tagged at the N-terminus with the minimum binding site for α-bungarotoxin (GABA_B2_(BBS)) were labeled with AlexaFluor 555 conjugated α-bungarotoxin and then analyzed by live cell imaging for internalization of labeled receptors. M1-Pep was added 10 min after starting the recording. Left: Representative images (scale bar: 5 μm). Right: Quantification of the immunofluorescence signal and statistical analysis. The statistical evaluation was performed on data recorded 15 min and 20 min after starting the recording. The data represent the mean ± SD of 7–8 neurons derived from three independent experiments. Unpaired t-test (ns, *p* > 0.05; *p < 0.05).

To further elucidate the function of MARCH1 on GABA_B_ receptors, we screened a set of synthetic overlapping peptides covering the C-terminal domains of GABA_B1_ and GABA_B2_ for their ability to inhibit the interaction of GABA_B_ receptors with MARCH1, thereby restoring MARCH1 induced downregulation of plasma membrane GABA_B_ receptors (Fig. 4a). All peptides contained 8 arginine at their N-terminus to render them cell permeable. The screening yielded 2 peptides, one comprising the GABA_B1_ sequence AEKEERVSELRHQLQSRQQL (1–4) and one comprising the GABA_B2_ sequence DKDLEEVTMQLQDTPEKTTY (2–5). However, the GABA_B2_ derived peptide (2–5) restored the downregulated GABA_B_ receptor more reliably than the GABA_B1_ derived peptide (1–4). This observation was substantiated in dose-response experiments of these two peptides (Fig. 4b). We found that 10 µg/ml peptide exerted the maximum effect on restoring GABA_B_ receptor expression with peptide 2–5 being more effective than peptide 1–4 (Fig. 4b).

Because the GABA_B2_ peptide sequence 2–5 is in proximity to the GABA_B_ receptor/CHOP interaction site^16,29^, we checked the specificity of peptide 2–5 by testing its ability to reverse CHOP-induced downregulation of plasma membrane GABA_B_ receptors in a co-transfection assay in HEK 293 cells. Peptide 2–5 did not restore the CHOP mediated downregulation of cell surface GABA_B_ receptors and therefore did not affect the interaction of CHOP with GABA_B_ receptors (Fig. 4c).

In all further experiments, we used peptide 2–5 tagged at the N-terminus with a peptide sequence derived from the Rabies virus glycoprotein (RVG), which renders it cell permeable in a neuron-specific manner via a receptor-mediated uptake mechanism^15,30,31^. The RVG-2-5 peptide is named M1-Pep in the rest of the study. In major experiments, the specificity of M1-Pep was verified against a control peptide (Ctrl-Pep). Ctrl-Pep consists of the RVG sequence followed by the 2–5 amino acids in a random sequence.

### M1-Pep did not affect cell surface expression of GABAB receptor under physiological conditions

To test whether a potential interaction of MARCH1 with GABA_B_ receptors affects receptor expression under physiological conditions, neuron/glia cultures were treated with M1-Pep and analyzed for GABA_B_ receptor expression (Fig. 4d). However, under this condition, treatment with M1-Pep at a concentration of 10 µg/ml neither affected cell surface nor total expression of GABA_B_ receptor. Thus, under physiological conditions GABA_B_ receptor expression may only marginally, if at all, affected by MARCH1.

Next, we tested for potential subtle effects of MARCH1 on constitutive internalization of GABA_B_ receptors. For this, neurons were transfected with GABA_B2_ tagged at the extracellularly located N-terminus with the minimum binding site for α-bungarotoxin (GABA_B2_(BBS)). After labeling cell surface receptors containing GABA_B2_(BBS) with AF555-conjugated α-bungarotoxin, neurons were analyzed by live cell imaging. Cell surface GABA_B_ receptors were continually reduced during the time span of the measurement (20 min) demonstrating constitutive internalization of the receptors (Fig. 4e). Adding M1-Pep after 10 min slightly reduced loss of cell surface receptors. However, this effect became statistically significant only after longer treatment times with M1-Pep (20 min, Fig. 4e).

### M1-Pep restored cell surface expression of GABAB receptors after OGD

**Fig. 5:**
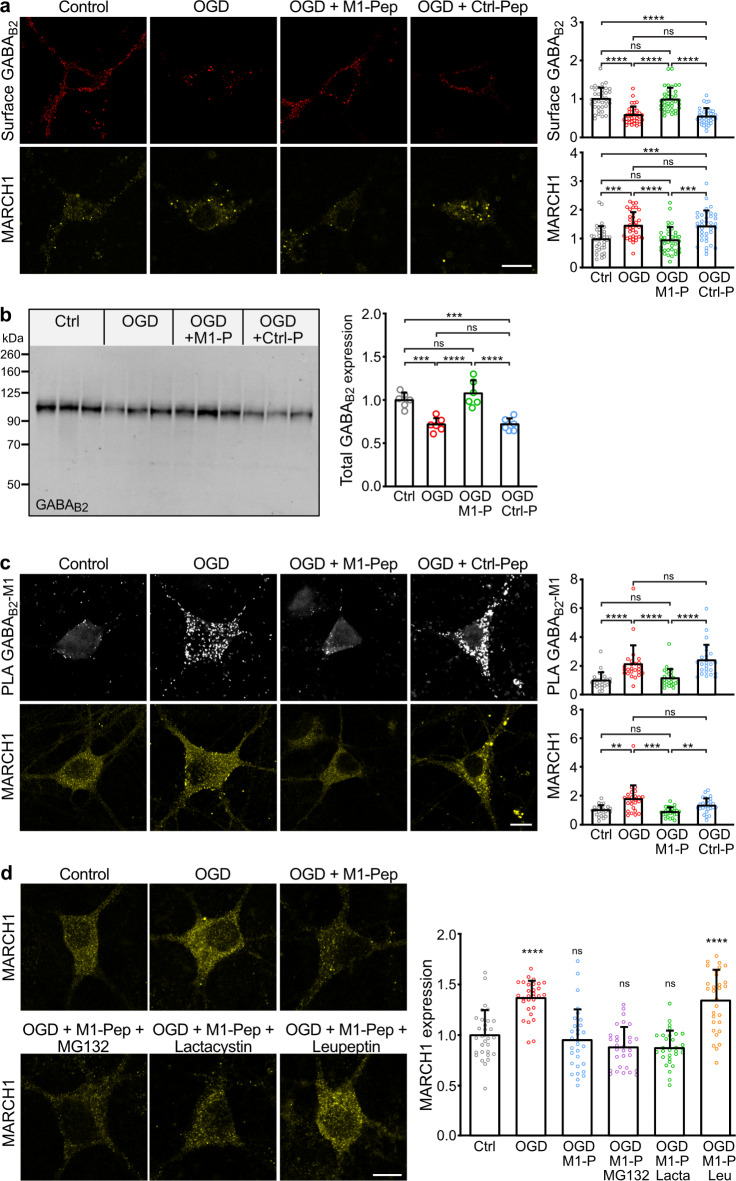
M1-Pep restored cell surface expression of GABA_B_ receptors after OGD by inhibiting the MARCH1/GABA_B_ receptor interaction. (**a**) Neurons/glia co-cultures were subjected to 1 h of OGD, then treated with M1-Pep or Ctrl-Pep for 16 h and analyzed for cell surface expression of GABA_B_ receptors and MARCH1 expression. Left: representative images (scale bar: 10 μm). Right: quantification of fluorescence intensities (mean ± SD of 36 neurons per condition, 3 independent experiments). One-way ANOVA followed by Tukey’s multiple comparison test (ns, *p* > 0.05; ***, *p* < 0.001; ****, *p* < 0.0001). (**b**) Western blot analysis of total GABA_B_ receptor expression using GABA_B2_ antibodies. Left: representative Western blot. Cultures were subjected for 1 h to OGD, then immediately treated or not with M1-Pep or Ctrl-Pep and harvested after 16–24 h for analysis. Right: quantification of Western blot signals (mean ± SD of 3 independent cultures per condition and one technical replicate). Signals were normalized to untreated control cultures (Ctrl). One-way ANOVA followed by Tukey’s multiple comparison test (ns, *p* > 0.05; ***, *p* < 0.001; ****, *p* < 0.0001). (**c**) M1-Pep inhibited the increased interaction between GABA_B_ receptors and MARCH1 after OGD as analyzed by in situ PLA using antibodies directed against GABA_B2_ and MARCH1. Signals were normalized to untreated cultures (Ctrl). Left: representative images; top images: in situ PLA signals (white dots), bottom images: MARCH1 staining (scale bar: 10 μm). Right: quantification of in situ PLA and MARCH1 signals (mean ± SD of 25 neurons per condition, 3 independent experiments). One-way ANOVA followed by Tukey’s multiple comparison test (ns, *p* > 0.05; ***, ****, *p* < 0.0001). (**d**) M1-Pep induced lysosomal degradation MARCH1. Cultures were subjected to OGD for 1 h and immediately thereafter remained untreated or were treated with M1-Pep in the presence of proteasomal inhibitors (50 µM lactacystin or 50 µM MG132) or the lysosomal inhibitor leupeptin (50 µM). Neurons were tested for MARCH1 expression. Left: representative images (scale bar: 10 μm). Right: quantification of fluorescence intensities (mean ± SD of 30 neurons per condition, 2 independent experiments). The fluorescence intensity of untreated neurons served as control. Brown-Forsythe and Welch’s one-way ANOVA followed by Dunnett’s T3 multiple comparisons test (ns, *p* > 0.05; ****, *p* < 0.0001).

Because MARCH1 was upregulated under ischemic conditions (Fig. 3b) we tested the effect of M1-Pep in restoring cell surface expression of GABA_B_ receptor after OGD. For this, we subjected neuron/glia cultures for 1 h to OGD stress followed by treatment with M1-Pep and determination of GABA_B_ receptor expression. OGD stress increased MARCH1 expression, as observed before, and concomitantly reduced cell surface expression of the receptors (Fig. 5a, Fig. S2). Treatment of cultures with M1-Pep after OGD normalized cell surface and total expression of GABA_B_ receptors (Fig. 5a, b). The OGD induced downregulation of GABA_B_ receptors was associated with an interaction of the receptors with MARCH1 as determined by in situ PLA. Under control conditions, we observed only few in situ PLA signals, most likely representing non-specific background (Fig. 5c). However, the PLA signals drastically increased after OGD stress, indicating the interaction of the receptors with MARCH1. Treatment of cultures with M1-Pep reduced PLA signals to control levels whereas the inactive Ctrl-Pep had no effect (Fig. 5c).

Interestingly, treatment of neuron/glia cultures with M1-Pep after OGD reduced the expression of MARCH1, suggesting that MARCH1 was degraded after binding to M1-Pep (Fig. 5c). To test for this, neuron/glia cultures were subjected to OGD, treated with M1-Pep and in addition with inhibitors for proteasomal (MG132, lactacyctin) or lysosomal (leupeptin) degradation (Fig. 5d). Inhibition of proteasomal degradation did not affect the downregulation of MARCH1, whereas blocking lysosomal degradation prevented it. Thus, binding of M1-Pep to MARCH1 most likely induced lysosomal degradation of a complex of M1-Pep bound to MARCH1.

**Fig. 6:**
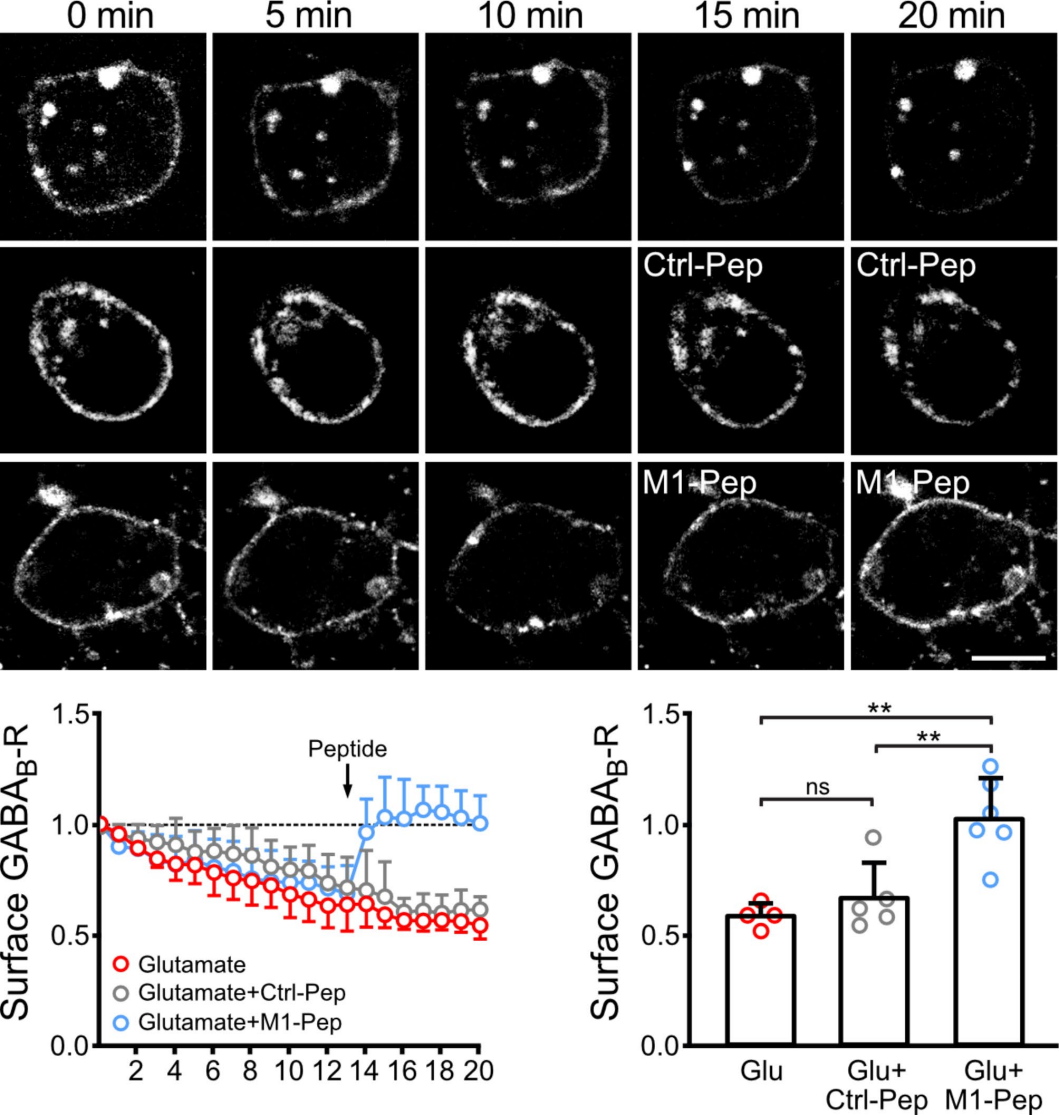
M1-Pep restored glutamate induced loss of cell surface GABA_B_ receptors. Neurons were transfected with GABA_B2_ tagged at the N-terminus with the minimum binding site for α-bungarotoxin (GABA_B2_(BBS)) together with EGFP (for quick identification of transfected neurons) and GABA_B1_. Cell surface receptors containing GABA_B2_(BBS) were labeled with AlexaFluor 555 conjugated α-bungarotoxin and then neurons were treated with 50 µM glutamate to induce downregulation of the receptors. Neurons were analyzed by live cell imaging for internalization of labeled receptors. M1-Pep and Crtl-Pep were added 13 min after adding glutamate. Top: Representative images (scale bar: 10 μm). Bottom: Quantification of the immunofluorescence signal and statistical analysis. The statistical evaluation was performed on data recorded 15 min after glutamate treatment. The data represent the mean ± SD of 4–6 neurons derived from three independent experiments. One-way ANOVA followed by Tukey’s multiple comparison test (ns, *p* > 0.05; **p < 0.01).

We hypothesized that MARCH1 downregulates cell surface expression of GABA_B_ receptors by permanently altering trafficking pathways of the receptor, such as internalization or recycling. If this is the case, M1-Pep should be able to quickly restore normal receptor expression. To test for how fast M1-Pep exerts its effect, we performed live cell imaging experiments as described for Fig. 4e, except that downregulation of cell surface receptors was induced by treating the cultures with glutamate. Cell surface GABA_B_ receptors were reduced to 64 ± 12% 13 min after glutamate application (Fig. 6). Adding M1-Pep after 13 min completely restored cell surface expression of the receptors (96 ± 15%) within 1 min. The Ctrl-Pep was without effect (70 ± 18%). This finding supports our hypothesis that MARCH1 affects trafficking of GABA_B_ receptors.

### M1-Pep increased fast recycling of GABA_B_ receptors

**Fig. 7:**
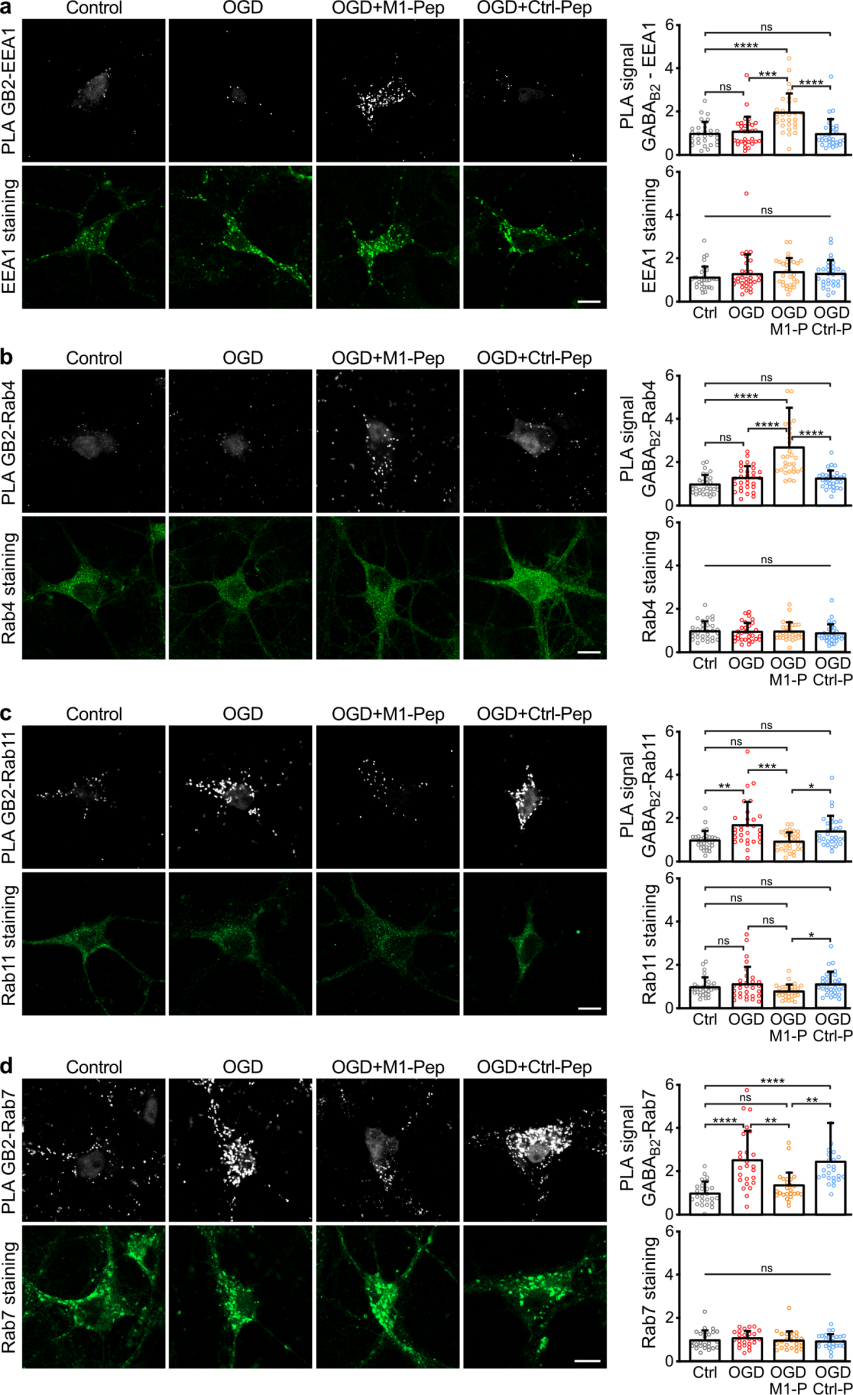
Treatment of OGD-stressed neurons with M1-Pep increased fast recycling of GABA_B_ receptors and inhibited their targeting to the lysosomal pathway. Cultures were subjected to OGD for 1 h followed by treatment with M1-Pep. Neurons were analyzed about 16 h after OGD for the colocalization of GABA_B_ receptors with endosomal markers by in situ PLA (upper panels) and for expression of the marker proteins (lower panels) using antibodies directed against GABA_B2_ and for the early endosome marker EEA1 (**a**), the fast recycling endosome marker Rab4 (**b**), the slow recycling endosome marker Rab11 (**c**), and the late endosome marker Rab7 (**d**). Scale bars, 10 µm. The data represent the mean ± SD of 25-30 neurons per condition derived from three independent experiments. One-way ANOVA followed by Tukey’s multiple comparison test (ns, p>0.05; *p<0.05; **p<0.01; ***p<0.001; ****p<0.0001).

To investigate the pathway underlying the M1-Pep induced restoration of cell surface GABA_*B*_ receptors after OGD, we analyzed the colocalization of the receptors with marker proteins for early endosomes (EEA1)^32^, fast recycling endosomes (Rab4)^33,34^, slow recycling endosomes (Rab11)^35^and late endosomes (Rab7)^36^using in situ PLA (Fig. 7).

Treatment of neuron/glia co-cultures with M1-Pep after OGD increased colocalization of GABA_B_ receptors with the marker proteins for early endosomes (EEA1, Fig. 7a) and for fast recycling endosomes (Rab4, Fig. 7b). In contrast, M1-Pep normalized the OGD induced increased colocalization of the receptors with slow recycling endosomes (Rab11, Fig. 7c) and late endosomes (Rab7, Fig. 7d). These results suggest that after an ischemic insult the treatment with M1-Pep restored GABA_B_ expression by increasing fast recycling of the receptors and thereby reducing the pathological sorting to lysosomal degradation.

### M1-Pep protects neurons from OGD stress induced death

**Fig. 8:**
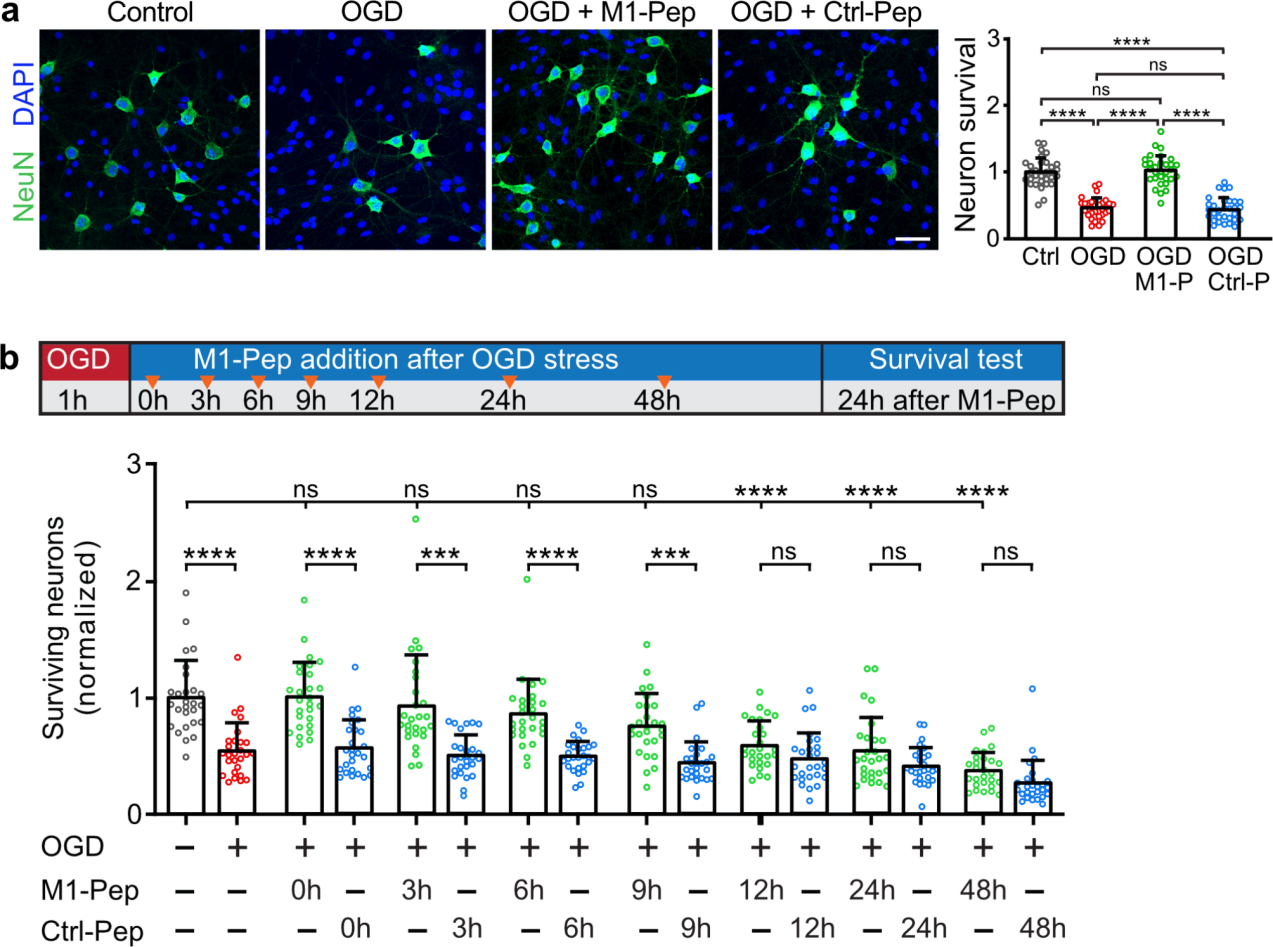
M1-Pep inhibits progressive neuronal death after OGD. (**a**) M1-Pep completely prevents neuronal death when added directly after OGD. Neuron/glia cultures were subjected to OGD for 1 h and immediately thereafter treated with M1-Pep or Crtl-Pep. Next day, the number of neurons was determined by immunostaining for the neuronal marker protein NeuN (green) and total cell number by DAPI staining (blue). Left: representative images (scale bar: 50 µm). Right: quantification of surviving neurons (ratio of neurons to total cells). Untreated cultures served as control. The data represent the mean ± SD of the analysis of 31 field of views per condition derived from three independent experiments. One-way ANOVA followed by Tukey’s multiple comparison test (ns, p>0.05; ****p<0.0001). (**b**) M1-Pep inhibited OGD induced progressive neuronal death. Neurons were subjected to OGD for 1 h and were then treated with M1-Pep-Pep or Ctrl-Pep at different time intervals. Twenty-four hours after peptide treatment, neurons were tested for the number of neurons and total cells. Top: scheme of experimental design. Bottom: quantification of surviving neurons. Untreated cultures served as control. The data represent the mean ± SD of the analysis of 27 field of views per condition derived from three independent experiments. Brown-Forsythe and Welch’s one-way ANOVA followed by Games-Howell’s multiple comparisons test (ns, p>0.05; ****p<0.0001).

Since M1-Pep restored the expression of GABA_B_ receptors after OGD, we tested its ability to limit excitotoxic neuronal death. For this, neuron/glia co-cultures were subjected for 1 h to OGD and then immediately treated with M1-Pep. Determination of the number of surviving neurons after 24 h revealed that M1-Pep completely prevented neuronal death whereas the control peptide showed no effect (Fig. 8a).

Next, we tested the neuroprotective efficacy of M1-Pep when added at different time intervals after OGD (0, 3, 6, 9, 12 and 24 h). The number of surviving neurons were counted after a further 24 h (Fig. 8b). M1-Pep inhibited progressive neuronal death when added up to 9 h after OGD (Fig. 8b). At later time points, the neuroprotective effect of M1-Pep was no longer visible due to the already massive death of neurons. Thus, restoring the GABA_B_ receptor expression by M1-Pep treatment after an ischemic insult limits progressing neuronal death within a wide time window.

## Discussion

**Fig. 9:**
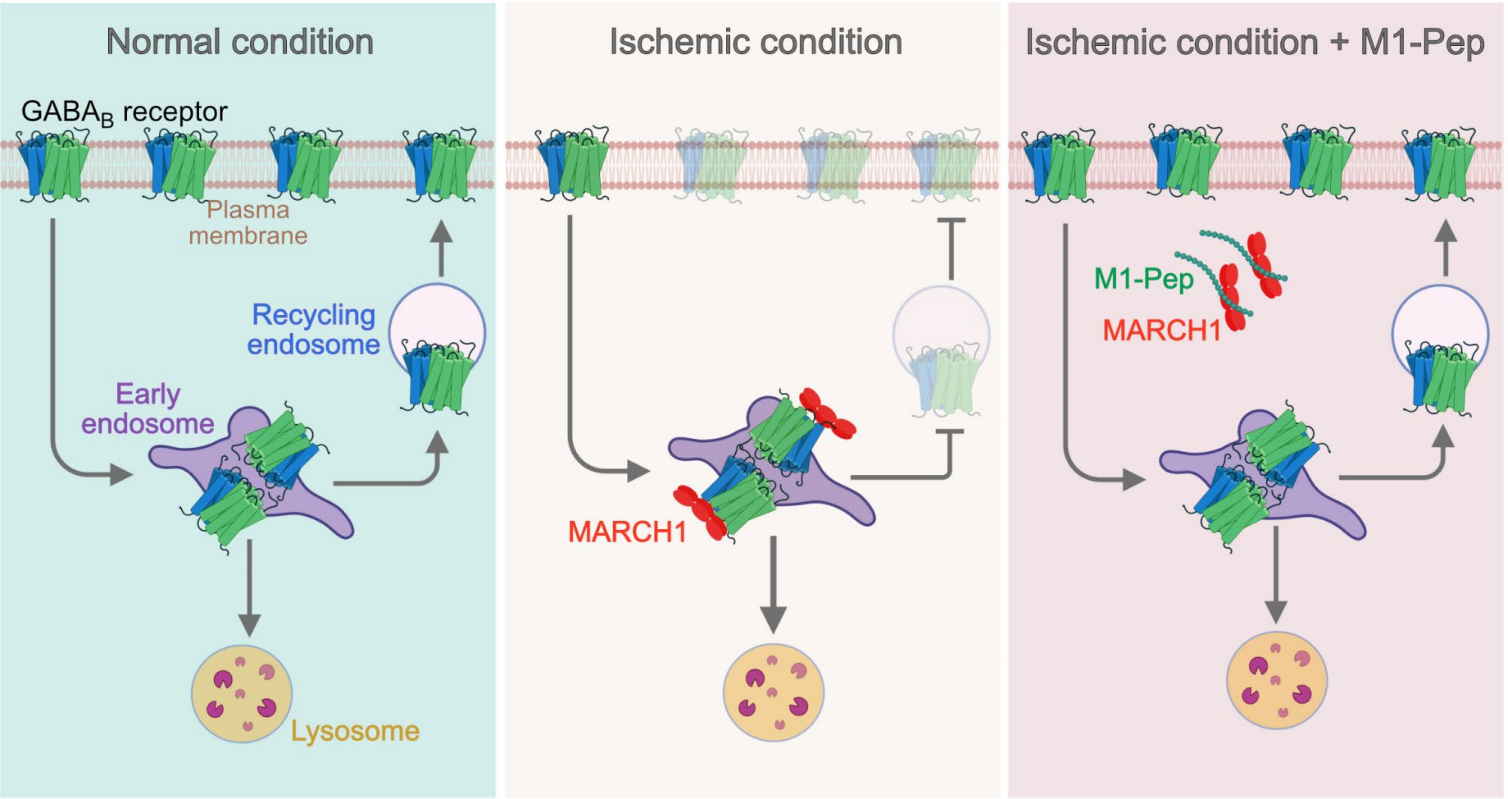
Proposed mechanism of MARCH1-induced downregulation of GABA_B_ receptors under ischemic conditions. Under normal physiological conditions, GABA_B_ receptors are constitutively endocytosed and most internalized receptors are recycled to the plasma membrane, whereas a fraction is degraded in lysosomes. Under ischemic conditions, upregulated MARCH1 interacts with GABA_B_ receptors and ubiquitinate them. This inhibits recycling of endocytosed GABA_B_ receptors. Blocking recycling causes rapid removal of GABA_B_ receptors from the plasma membrane and triggers their lysosomal degradation. Application of M1-Pep after ischemic stress inhibits the interaction of MARCH1 with GABA_B_ receptors. This re-establishes recycling of the receptors to the plasma membrane and normalizes enhanced lysosomal degradation of GABA_B_ receptors, resulting in normal plasma membrane expression of GABA_B_ receptors and inhibition progressive neuronal death. The figure was created using https://www.biorender.com/.

In this study, we explored for membrane associated E3 ubiquitin ligases that are involved in regulating cell surface expression of GABA_B_ receptors specifically under pathological conditions. We identified MARCH1 as a critical element in downregulating plasma membrane expression of GABA_B_ receptors under ischemic/excitotoxic conditions (Fig. 9). MARCH1 (also known as RNF171) is a transmembrane E3 ligase, whose mRNA is particularly well expressed in secondary lymphoid tissues (spleen and lymph nodes) and at lower levels in other tissues including the brain^25,26^. MARCH1 has been shown to downregulate cell surface expression of MHC class II molecules, CD86, transferrin receptors and insulin receptors by targeting them to lysosomal degradation^19,24,26,37,38^.

We found that co-expression of GABA_B_ receptors with MARCH1 in HEK-293 cells downregulated the cell surface receptors and required - in addition to the association of MARCH1 with the heterodimeric receptor - intact ubiquitin ligase activity, i.e. ubiquitination of the receptor. So far, it has been shown that targeting of GABA_B_ receptors to lysosomal degradation involves Lys63-linked polyubiquitination of least three lysine residues in GABA_B1_ by the E3 ligase Mind Bomb-2 ^39,40^. In addition, long-term changes in neuronal activity alters the level of cell surface GABA_B_ receptors by regulating the number of newly synthesized receptors in the ER via Lys48-linked polyubiquitination of the receptors by the ER E3 ligase Hrd1 and proteasomal degradation^41,42^. Here, we found that MARCH1 is not involved in Lys48- or Lys63-linked polyubiquitination of GABA_B_ receptors but most likely in mono- or mult-monoiubiquitination of GABA_B_ receptors at yet undefined sites. This is in line with reports that implicated MARCH 1 in mono-, Lys63- and Lys48-linked polyubiquitination of target proteins^43–46^.

For gaining more insights into the mechanism of MARCH induced downregulation of GABA_B_ receptors, we developed an interfering peptide (M1-Pep) inhibiting the interaction of the receptors with MARCH1. Treatment of cultured neurons with M1-Pep did not appreciably affect cell surface expression of the receptor under physiological conditions. This finding was not surprising in view of the very low expression of MARCH1 protein reported in primary cells^19,44,47^ and the lack of interaction we detected under physiological conditions. However, in live cell imaging experiments, testing for a potential effect of MARCH1 on constitutive internalization of GABA_B_ receptors, we observed a slightly lower loss of cell surface receptors after prolonged incubation with M1-Pep. It is very unlikely that this effect is related to a direct action of MARCH1 on internalization of the receptors. We rather attribute this observation to an upregulation of MARCH1 under the suboptimal culture conditions during microscopy, which might induce cellular stress at longer time points.

Only after upregulation of MARCH1 by subjecting neurons to excitotoxic or ischemic stress, GABA_B_ receptors significantly interacted with MARCH1 and robustly downregulated plasma membrane expression of the receptors. It is well established that MARCH1 is largely localized to the endocytic pathway where MARCH1 does not affect internalization of target proteins but triggers their lysosomal degradation^26,38,48^. In our experiments, co-expression of GABA_B_ receptors with MARCH1 in HEK293 cells solely downregulated cell surface receptors but not total receptor levels arguing against a direct involvement of MARCH1 in targeting the receptors to lysosomal degradation. Instead, MARCH1 appears to inhibit fast recycling of the receptors, as concluded from colocalization experiments of the receptors with marker proteins of the endocytic pathway in neurons subjected to ischemic stress. Inhibition of the MARCH1/GABA_B_ receptors interaction by M1-Pep restored fast recycling of the receptors and as a consequence also normalized enhanced lysosomal degradation of the receptors. This effect restored normal cell surface expression levels of the receptors and thereby prevented ischemia-induced neuronal death.

Interestingly, enhanced MARCH1 expression in ischemia-stressed neurons was normalized to control levels by treatment with M1-Pep. MARCH1 has a short half-life of less than 30 min^47^ and its expression is tightly controlled by dimerization and trans-autoubiquitination^44^. Both, lysosomes and proteasomes have been implicated in the degradation of MARCH1 ^44,47^. In our setting, inhibition of lysosomes prevented downregulation of MARCH1 by M1-Pep, whereas blockers of proteasomes were ineffective. Thus, under ischemic conditions, the upregulated MARCH1 presumably forms a complex of MARCH1 with M1-Pep and is selectively degraded by lysosomes. Whether or not an interfering peptide induces degradation of its target protein appears not to be easily predictable. The interfering peptides we previously developed to inhibit the interaction of GABA_B_ receptors with CaMKIIβ^15^ and PP2A^12^ did not affect the expression levels of CaMKIIβ and PP2A, whereas the peptide interfering with the CHOP/GABA_B_ receptor interaction induced proteasomal degradation of ischemia-mediated upregulated CHOP^16^. The similar properties of MARCH1 and CHOP are the tight regulation of their expression (they are marginally expressed under physiological conditions^19,47,49–51^) and their detrimental effect on cellular health when overexpressed. Hence, tightly regulated proteins that interfere with cellular health at higher expression levels might be more susceptible to degradation when interacting with an interfering peptide.

We have previously shown that restoring GABA_B_ receptor expression after an ischemic insult using interfering peptides targeting the CaMKIIβ^15^, PP2A^12^, or CHOP^16^ interaction with the receptor re-established normal GABA_B_ receptor mediated inhibition. This inhibited enhanced excitability of affected neurons and prevented progressing neuronal death. These peptides are promising candidates for the development of a neuroprotective treatment in the acute phase of stroke. However, the interfering peptides targeting CaMKIIβ and PP2A have the potential limitation that they inhibit interactions that are regulating GABA_B_ receptor expression under physiological conditions as well. Treatment with these peptides could theoretically increase GABA_B_ receptor expression in non-diseased neurons. By contrast, the interfering peptides inhibiting the interaction of the receptors with CHOP or MARCH1 target interactions that are not occurring under physiological conditions and require disease-induced upregulation of MARCH1 and CHOP. Thus, the interaction of CHOP and MARCH1 with GABA_B_ receptors are specifically occurring in diseased neurons. Therefore, treatment with the respective interfering peptides should not affect non-diseased neurons and are expected to exhibit no or minimal side effects related to GABA_B_ receptors. In a next step, the neuroprotective activity of CHOP and MARCH1 interfering peptides need to be tested in suitable animal models of cerebral ischemia.

## Methods

### Plasmids

The following plasmids were used for this study: hemagglutinin (HA)-tagged GABA_B1a_^52^; GABA_B2_^53^; GABA_B1a_(RSAR)^54^; EYFP-MARCH1 ^18^; EYFP-MARCH1(DN)^18^; EYFP-MARCH1-KKxx^24^; EYFP-MARCH5 ^18^; EYFP-MARCH5(DN)^18^; EYFP-MARCH8 ^24^; EYFP-MARCH8(DN)^24^; EYFP-RNF112 ^18^; EYFP-RNF112(DN)^18^; EYFP-RNF133 ^18^; EYFP-RNF133(DN)^18^; EYFP-RNF144 ^18^; EYFP-RNF144(DN)^18^; EYFP-RNF152 ^18^; EYFP-RNF152(DN)^18^; EYFP-RNF167 ^18^; EYFP-RNF167(DN)^18^; CHOP^29^; ubiquitin(KO) (Addgene plasmid 17603 ^55^), and HA-GABA_B2_(BBS) tagged the minimum α-bungarotoxin binding site (BBS)^12^.

### Drugs

The following drugs were used for this study: MG-132 (Abcam, #ab141003), lactacystin (Abcam, #ab141411), leupeptin (Merck, #L2884), D-tubocurarine chloride (Abcam, #AB120073) and AlexaFlour 555-conjugated α-bungarotoxin (Invitrogen, #B35451).

### Antibodies

The following antibodies were used for this study: mouse anti-MARCH1 (1:250 for Western blotting [WB], 1:250 for immunofluorescence staining [IF] and 1:50 for in situ proximity assay [PLA], Sigma Aldrich #WH0055016M2), rabbit anti-MARCH1 (1:250 for IF and 1:50 for PLA, Antibodies Online, #ABIN2705354), mouse anti-GABA_B1_ (1:250 for IF and, 1:50 for PLA; Abcam #ab55051), rabbit anti-GABA_B1b_ directed against the N-terminus of GABA_B1b_ (affinity-purified,1:100 for IF; custom made by GenScript^56^), rabbit anti-GABA_B2_ directed against the N-terminus of GABA_B2_ (affinity-purified, used for cell surface staining, 1:250 for IF; custom made by GenScript^57^), rabbit anti-GABA_B2_ (1:500 for IF, 1:100 for PLA, 1:800 for WB; Abcam #ab75838), rabbit anti-NeuN (1:400 for IF, Millipore #ABN78), rabbit anti-HA (1:500 for IF and 1:50 for PLA, Sigma-Aldrich #SAB5600116), rabbit anti-ubiquitin-Lys63 (1:50 for PLA, Millipore #05-1307), rabbit anti-ubiquitin-Lys48 (1:50 for PLA, Millipore #05-1308), mouse anti-EEA1 (1:50 for PLA, BD Biosciences #610456), mouse anti-Rab4 (1:50 for PLA, BD Biosciences #610888), mouse anti-Rab11 (1:50 for PLA, Millipore #05-853), and rabbit anti-Rab7 (1:50 for PLA, Abcam #ab137029). For immunofluorescence staining, secondary antibodies used were labeled with either Alexa Fluor Plus 488, 555, or 647 (1:2000, ThermoFisher). For Western blotting secondary antibodies were conjugated to IRDye800CW (LI-COR Biosciences).

### Culture and transfection of HEK-293 cells

HEK-293 cells (Human Embryonic Kidney, ATCC) were cultured in DMEM (Gibco Life Technologies) containing 10% fetal bovine serum (FBS) (Gibco Life Technologies) and penicillin/streptomycin (Gibco Life Technologies). HEK-293 cells were plated at a density of 100,000 cells per ml into 12 well plates containing poly-D-lysine coated coverslips and kept overnight in incubator at 37 °C and 5% CO_2_. Next day, the cells were transfected with respective plasmids using the polyethyleneimine (PEI) method according to the jet-PEI protocol (Polyplus Transfection) and then kept in incubator at 37 °C and 5% CO_2_ for 24–48 h.

### In situ proximity ligation assay (in situ PLA)

The in situ PLA was performed using the Duolink II kit (Sigma Aldrich) according to the instructions of the manufacturer. Briefly, the neurons or HEK-293 cells were washed for 5 min with PBS and then fixed with 4% PFA for 30 min at room temperature. Then, the coverslips were rinsed in PBS for 5 min and permeabilized for 15 min with 0.2% Triton X-100/PBS. After rinsing the coverslips in PBS, they were incubated with two primary antibodies between whom the interaction had to be observed (one antibody raised in rabbit and other in mouse) overnight in a humidity chamber at 4 °C. Subsequently, the cultures were washed four times for 5 min with PBS and incubated for 30 min at room temperature with the PLA probes (prepared by diluting anti-Mouse MINUS and anti-Rabbit PLUS (Duolink II) at a concentration of 1:5 in 10% NGS/PBS). Afterwards, 60 µl of the PLA probe solution were pipetted on top of each coverslip and incubated in a humidity chamber at 37 °C for 1 h. The coverslips were then washed in PBS two times for 5 min and then incubated at 37 °C for 30 min with ligation solution. Subsequently, the cells were washed two times in Duolink II Wash Buffer A and then incubated with the amplification solution at 37 °C for 100 min. Finally, the coverslips were washed two times for 10 min with Duolink II Wash Buffer B in the dark and then mounted onto microscope slides with DAKO fluorescent mounting medium.

### Primary neuron-glia co-cultures

In this study, we used mixed neuron/glia co-cultures because they mimic the brain physiology more closely compared to the pure neurons. The neuron to glial cell ratio in our cultures was roughly 1:5. All procedures were carried out according to the national guidelines of the Swiss Federal act on animal protection and were approved by the Cantonal Veterinary Office Zurich (license ZH011/19 and ZH087/2022). We confirm compliance with the ARRIVE guidelines.

Pregnant Wistar rats were purchased from ENVIGO, Netherlands. All cell culture media used were from Gibco. The pregnant rat was deeply anesthetized with isoflurane, euthanized by decapitation and embryos were extracted for preparation of primary cultures. Embryos (18 days old) were killed by decapitation and the cerebral cortices were dissected and then washed with 5 ml sterile-filtered PBGA buffer (PBS containing 10 mM glucose, 1 mg/ml bovine serum albumin and antibiotic-antimycotic 1:100 (10,000 units/ml penicillin; 10,000 µg/ml streptomycin; 25 µg/ml amphotericin B). The cortices were cut into small tissue pieces with a sterile scalpel and then digested by incubating in 5 ml sterile filtered papain solution for 15 min at 37 °C. The supernatant was then removed and the tissue was washed twice with complete DMEM/FBS medium (Dulbecco’s Modified Eagle’s Medium containing 10% Fetal Bovine Serum and penicillin/streptomycin, 1:100). Then, 3–4 ml of fresh DMEM/FBS was added, and the tissue was carefully and gently triturated and subsequently filtered through a 40 μm cell-strainer. Finally, the neurons were plated at a density of 75,000–90,000 cells per well onto the poly D-lysine (Gibco Life Technologies) coated coverslips in a 12-well culture dish and incubated for 3–4 h at 37 °C and 5% CO_2_. Then, the DMEM medium was exchanged with freshly prepared NU-medium (Minimum Essential Medium (MEM) with 15% NU serum, 2% B27 supplement, 15 mM HEPES, 0.45% glucose, 1 mM sodium pyruvate, 2 mM GlutaMAX). The cells were kept in incubator for 12–16 days at 37 °C and 5% CO_2_.

### Transfection of primary Neuron/Glia cultures

After 7 to 12 days in vitro cultures were transfected with a total of 1 µg of the respective plasmid DNAs by the magnetofection method using Lipofectamine 2000 (Invitrogen) and CombiMag (OZ Biosciences) at 37 °C and 5% CO_2_, as described by Buerli et al.^58^.

### Oxygen and glucose deprivation (OGD) stress

Neuronal cultures were subjected to OGD stress by exposure to OGD medium (DMEM lacking glucose, glutamine, sodium pyruvate, HEPES and phenol red) in a hypoxic incubator. The OGD medium was deprived from oxygen by equilibrating it with nitrogen for 15 min in a water bath at 37 °C. For IF, 1 ml of equilibrated OGD medium was added into each well of a 12-well culture plate and the coverslips containing the cultured neurons were transferred to the OGD medium. The culture plate was then incubated for 1 h in a hypoxic incubator at 1% O_2_, 5% CO_2_ and 37 °C. The coverslips were then transferred back to the culture plate containing the original conditioned culture medium and incubated at 37 °C and 5% CO_2_. For WB, 5 ml of equilibrated OGD medium was added into each 6 cm dishes followed by 1 h in a hypoxic incubator at 1% O_2_, 5% CO_2_ and 37 °C. The equilibrated OGD medium was then removed and 5 ml of original conditioned culture medium was added and incubated at 37 °C and 5% CO_2_. Unless stated otherwise, the neurons were analyzed after a recovery period of 16–24 h.

### Interfering peptide (M1-Pep)

For identification of peptides interfering with the GABA_B_ receptor/March1 interaction a small library of overlapping synthetic peptides (15–25 amino acids long, for sequences see Fig. 4) comprising the intracellularly located C-terminal amino acid sequences of GABA_B1_ and GABA_B2_ was generated. All peptides contained a sequence of eight arginine at their N-terminus to render them cell permeable. To enhance peptide uptake into transfected HEK-293 cells, the cultures were washed three times with PBS and then treated with 50 µM pyrene butyric acid for 5 min. After three additional washes with PBS, the cells were treated with 10 µg/ml peptide and tested for their ability to inhibit the downregulation of plasma membrane GABA_B_ receptors.

From this screening resulted the interfering peptide DKDLEEVTMQLQDTPEKTTY (M1-Pep). For control, a peptide (Ctrl-Pep, QTDTYKMLDQELTTDPEEVK) was used which contains the same amino acids as M1-Pep but in a random manner. The cell permeability of the peptides was achieved by tagging the N-terminus of the respective peptide with a peptide sequence from Rabies virus glycoprotein followed by 9 arginine (RVG, YTIWMPENPRPGTPCDIFTNSRGKRASNGGGGRRRRRRRRR)^30^. The Peptides were custom-synthesized by Pepmic Co., Ltd, Suzhou, China. The peptides were used at a concentration of 10 µg/ml for all the experiments.

### Immunofluorescence staining

For cell surface staining of GABA_B_ receptors, an antibody directed against the N-terminus of GABA_B2_ (GABA_B2N_) was used. Coverslips containing the cultured neuron/glia cells were washed 3 times with cold buffer A (25 mM HEPES pH 7.4, 119 mM NaCl, 2.5 mM KCl, 2 mM CaCl_2_, 1 mM MgCl_2_ and 30 mM glucose). Then, the GABA_B2N_ antibody (1: 250 dilutions in buffer A containing 10% normal donkey serum (NDS)) was added and incubated on ice for 90 min. The coverslips were subsequently washed 3 times for 5 min with buffer A, followed by incubation with Alexa Fluor Plus anti-rabbit secondary antibody (1:2000 in PBS/10% NDS) for 60 min on ice. Afterwards, the coverslips were washed again 3 times for 5 min with buffer A. For subsequent staining of total MARCH1, the cells were fixed with 4% PFA for 30 min at room temperature. After fixation, the cells were washed with PBS and permeabilized by incubation for 12 min in 0.2% Triton X-100/PBS. Then, MARCH1 antibody (1:250 in PBS/10% NDS) was added and incubated overnight at 4 °C. After incubation, the coverslips were washed 3 times for 5 min with PBS. Then, Alexa Fluor Plus anti-mouse secondary antibody (dilution 1:2000 in PBS/ 10% NDS) was added and incubated for 1 h at room temperature. Finally, the coverslips were washed again 3 times for 5 min with PBS and mounted in DAKO fluorescence mounting medium onto glass slides for confocal microscopy.

For standard immunofluorescence staining, the cultures were briefly washed in PBS and then fixed with 4% PFA for 30 min at room temperature. After fixation, the cells were washed with PBS and permeabilized by incubation for 12 min in 0.2% Triton X-100/PBS, followed by incubation with primary antibody (diluted in PBS/10% NDS) overnight at 4 °C. Next day, the coverslips were washed 3 times for 5 min with PBS and incubated for 1 h at room temperature with Alexa Fluor Plus secondary antibody (diluted in PBS/ 10% NDS). The coverslips were then washed again 3 times for 5 min with PBS and mounted in DAKO fluorescence mounting medium onto glass slides for confocal microscopy.

### Live cell imaging for tracing internalization of GABA_B_ receptors

The live cell imaging of GABA_B_ receptors tagged with the α-bungarotoxin binding site (BBS) was performed for monitoring the internalization of the receptor exactly as described previously^12^. Neuron/glia cultures were transfected at DIV 7–12 with GABA_B2_(BBS) together with EGFP (for fast identification of transfected neurons). For live cell imaging, the cultures were washed with ice-cold Krebs solution (140 mM NaCl, 4.7 mM KCl, 1.2 mM MgCl_2_, 2.5 mM CaCl2, 11 mM glucose, and 5 mM HEPES pH 7.4), pre-incubated with 1 mM D-tubocurarine for 5 min to block endogenous nicotinic acetylcholine receptors containing the α7 subunit and then incubated on ice with 3 µg/ml AlexaFlour 555-conjugated α-bungarotoxin for 15 min to label the GABA_B_ receptors containing GABA_B2_(BBS) expressed at the cell surface. Then, the cultures were washed with ice-cold Krebs solution to remove excess BTX and transferred to the imaging chamber with warm Krebs solution at room temperature to enable internalization of the receptors. The EGFP expressing cells were located as quickly as possible, and settings were optimized for acquiring BTX florescence at time zero (t0). The imaging of a single plane was performed at Nyquist sampling and eight times averaging to increase the signal/noise ratio. Glutamate (50 µM) was added at the start of the recording and M1-Pep (10 µg/ml) 13 min thereafter. Both were added directly to the imaging chamber. The rate of GABA_B_ receptor internalization was evaluated by measuring the mean fluorescence intensity at the cell surface at every time-point. Measurements were normalized to the mean cell surface membrane fluorescence obtained at t0 time point.

### Quantification of neuronal loss

Neuron-glia co-cultures were subjected to OGD and subsequently treated with M1-Pep or control peptide. Then, cultures were incubated with an antibody directed against the neuron-specific marker protein NeuN (1:400 in PBS/ 10% NDS), followed by staining with Alexa Fluor Plus 488 secondary antibodies (1:2000 in PBS/ 10% NDS). The total number of cells were determined by counting the cell nuclei stained with DAPI included in the fluorescent mounting medium. After Microscopy, the neuronal loss was counted by ratio of number of neurons over number of DAPI positive cells using ImageJ plugin “Cell Counter”.

### Western blotting

For Western blot analysis of neuron-glia co-cultures, the cells were grown for 12–16 days on 6 cm poly-D lysine coated culture dishes at a density of 500,000 cells per dish. The cultures were washed two times with ice-cold PBS, harvested, and homogenized by sonication. The samples were incubated with Laemmli sample buffer (Bio-Rad) for 1 h at 37 °C and aliquots containing 20 µg protein were subjected to sodium dodecyl sulfate-polyacrylamide gel electrophoresis (SDS-PAGE) using 7.5% mini-gels (Mini Protean3; Bio-Rad). The proteins were transferred onto nitrocellulose membranes in a semi-dry transfer cell (Trans-Blot SD; Bio-Rad) at 15 V for 75 min. After blotting, the transferred total proteins were stained with REVERT 700 Total Protein Stain (LI-COR Biosciences) and detected by the ODYSSEY CLx scanner (LI-COR Biosciences). The blots were blocked for 1 h in PBS containing 5% nonfat dry milk at room temperature, followed by incubation with primary antibody overnight at 4 °C in PBS containing 5% nonfat dry milk. The blots were then washed five times for 5 min with TBST and incubated with secondary antibodies for 1 h at room temperature. The blots were washed again with TBST and immunoreactivity was detected by the ODYSSEY CLx scanner (LI-COR Biosciences). Immunoreactivity was quantified with the Image Studio software (LI-COR Biosciences) and normalized to total protein in the corresponding lanes.

### Microscopy and image analysis

Images were taken with a Zeiss laser scanning confocal microscope (CLSM800 AiryScan) in the sequential mode using the Zeiss 40 × (1.3 NA) or 63 × (1.45 NA) Plan-Fluor objective. Signal saturation was avoided by adjusting the values of laser intensity and the detector gain accordingly. All images of one experiment was imaged in one continuous session with the same settings. The images were quantitatively analyzed using ImageJ (v1.54i). For quantification of cell surface staining, the outer and inner perimeter of the cell surface were exactly outlined. Then, the fluorescence intensity value obtained from the inner border was subtracted from the one of the outer border so that only the fluorescence present at the cell surface was determined and used for statistical evaluation. For quantification of the total cell staining, only the outer border of the cell was marked and the mean fluorescence intensity was measured. For quantification of in situ PLA signals, the soma of neurons or HEK-293 cells was surrounded and the fluorescent dots inside these borders were counted using the ImageJ option “Find maxima”. A fixed noise tolerance value was used for the analysis of all images of the same experiment. The PLA signals were normalized to the area analyzed and also normalized to the GABA_B_ receptor expression level.

### Statistics

The statistical evaluation of data was performed using the software GraphPad Prism (version 8.4.3). Results were given as mean value ± standard deviation (SD). All data sets were tested for normal or lognormal distributions and analyzed by One-Way ANOVA followed by appropriate post hoc tests or unpaired t-test. Data sets that did not show normal distribution were analyzed by the Kruskal-Wallis test. In case of significant deviation from homoscedasticity, Welch and Brown Forsythe variations of ANOVA was used. A p-value of < 0.05 was considered as statistically significant. Details of the statistics are given in the figure legends.

## Electronic supplementary material

Below is the link to the electronic supplementary material.

Supplementary Material 1.

Supplementary Material 2.

## Data Availability

The datasets analyzed for this study are deposited at ZENODO.org and are publicly available as of the date of publication (10.5281/zenodo.14608218). Due to their large size raw images can be made available only upon reasonable request to the corresponding author.
