# Supplementary figures and images for: The E3 ubiquitin ligase MARCH1 mediates downregulation of plasma membrane GABAB receptors under ischemic conditions by inhibiting fast receptor recycling
Source: Sci Rep. 2025 Jan 8;15:1330. doi: 10.1038/s41598-025-85842-1 (PMC11711762; doi:10.1038/s41598-025-85842-1)

# Supplementary figure 1

Full size Western blot shown in Fig. 5 b

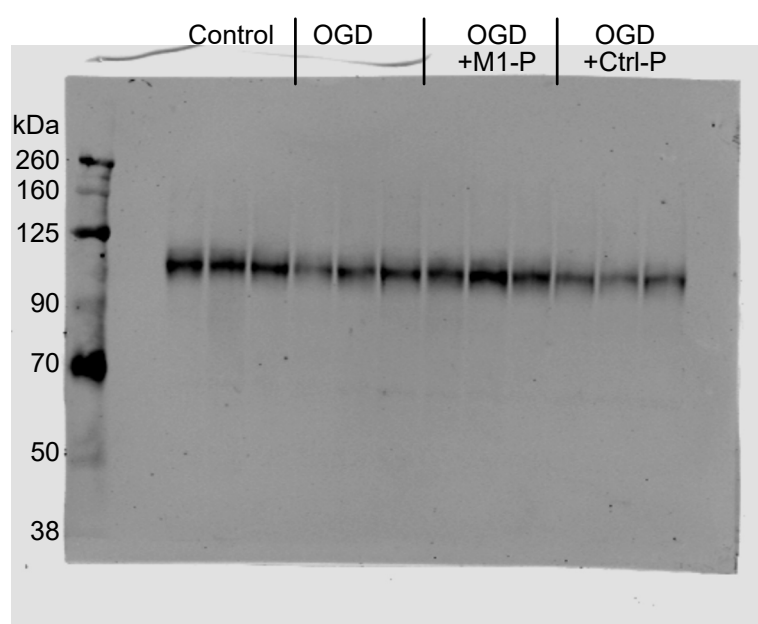

Supplement: Supplementary file 1 — Supplementary Material 1. [file 41598_2025_85842_MOESM1_ESM.pdf]
