# Supplementary material for: The E3 ubiquitin ligase MARCH1 mediates downregulation of plasma membrane GABAB receptors under ischemic conditions by inhibiting fast receptor recycling
Source: Sci Rep. 2025 Jan 8;15:1330. doi: 10.1038/s41598-025-85842-1 (PMC11711762; doi:10.1038/s41598-025-85842-1)
Supplement: Supplementary file 2 — Supplementary Material 2. [file 41598_2025_85842_MOESM2_ESM.pdf]

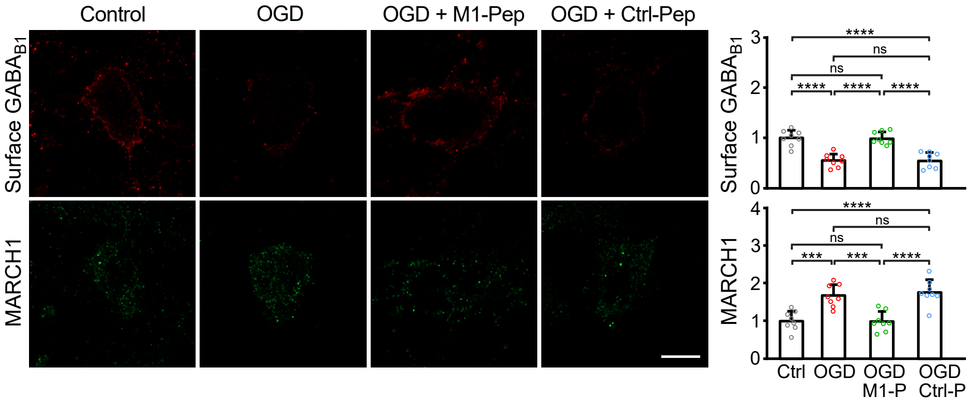

Supplementary Figure 2. M1-Pep restored cell surface expression of $GABA_B$ receptors after OGD by inhibiting the MARCH1/$GABA_B$ receptor interaction. (**a**) Neurons/glia co-cultures were subjected to 1 h of OGD, then treated with M1-Pep or Ctrl-Pep for 16 h and analyzed for cell surface expression of $GABA_B$ receptors and MARCH1 expression using antibodies directed against $GABA_{B1}$ and MARCH1. Left: representative images (scale bar: 10 µm). Right: quantification of fluorescence intensities (mean ± SD of 8 neurons per condition, 2 independent experiments). One-way ANOVA followed by Tukey's multiple comparison test (ns, $p > 0.05$; ***, $p < 0.001$; ****, $p < 0.0001$).
